# COVID-19 Management in Iran as One of the Most Affected Countries in the World: Advantages and Weaknesses

**DOI:** 10.3389/fpubh.2020.00510

**Published:** 2020-09-15

**Authors:** Maryam Rassouli, Hadis Ashrafizadeh, Azam Shirinabadi Farahani, Mohammad Esmaeil Akbari

**Affiliations:** ^1^Cancer Research Center, Shahid Beheshti University of Medical Sciences, Tehran, Iran; ^2^Student Research Committee, Nursing & Midwifery School, Ahvaz Jundishapur University of Medical Sciences, Ahvaz, Iran; ^3^Department of Pediatric & Neonatal Intensive Care Nursing, School of Nursing & Midwifery, Shahid Beheshti University of Medical Sciences, Tehran, Iran

**Keywords:** coronavirus, COVID-19, health policy, management report, Islamic Republic of Iran

## Abstract

COVID-19 management is a hot topic due to its extensive spread across the world and the declaration of pandemic status. How a crisis is managed in each country is influenced by several factors, and various strategies are applied in accordance with these factors in order to manage the crisis. Due to the rapid spread and increasing trend of the crisis and the fact that almost more than half of the countries are engaged in this pandemic, it is impossible to apply trial-and-error based strategies. One of the best strategies is to use the experiences of other countries in dealing with COVID-19. This report explores the advantages and weaknesses of the Islamic Republic of Iran in the management of this crisis in regard with political economic and cultural issues, health service coverage, and the transparency of information that can be used as a model for other countries around the world.

The Islamic Republic of Iran, as the second country to declare two deaths due to coronavirus, within 50 days after China on February 18, 2020 (1), is still one of the countries to deal with most cases of COVID-19 infection and the subsequent deaths (2). Obviously, managing the disease, which is considered a pandemic according to the World Health Organization (3), requires specific strategies that may vary due to different factors in each country, which may either lead to effectively dealing with the disease or cause challenges.

Considering the fact that using global experience, especially in times of crisis, is one of the best crisis management mechanisms, a review of the strengths and weaknesses of the Islamic Republic of Iran in the COVID-19 management covering the political-economic aspects, Health services coverage, cultural aspect, and the transparency of information can be used as a model by other involved countries, while at the same time benefiting from the strategies of countries with similar experiences.

Health is not only a biological, but also a political, social, cultural, and economic issue. “Health is a political issue” has been a point of consensus for a long time (4). Therefore, the ability of countries to manage COVID-19 is strongly influenced by their political-economic conditions which can be considered both an advantage and a threat. Thus, it can be said that sanctions as a political-economic factor, more than any other factor, have challenged Iran's ability to cope with COVID-19. COVID-19 spreads in Iran at the same time as the most severe sanctions are imposed on Iran. Although over the past four decades various sanctions have always been imposed on Iran, since May 2019, the unilateral US sanctions against Iran have been increased significantly (5). The Iranian health system has been directly and indirectly impacted by these sanctions, although it is one of the most prominent health systems in the Eastern Mediterranean Region (6). Although it is believed that sanctions are imposed on the physical weapons of war and do not include medicines and medical equipment, due to difficulties in commercial and financial exchanges with most countries, some essential medicines and laboratory equipment especially diagnostic, medical, and protection kits are not sufficiently available. In addition, numerous sanctions in the field of publishing research articles impede the international community's awareness of the consequences of such sanctions.

On the other hand, as an advantage, the influential presence of effective and socially acceptable positions such as the Iranian Supreme Leader as the highest religious authority and commander-in-chief can be named which helped in facing many unbearable challenges rooted in the beliefs, the culture and the religion of Iranian people, by taking measures such as ordering the General Staff of the Armed Forces to assist with the implementation of the regulations made by the Supreme National Security Council, thanking the medical community on many occasions, advising on the implementation and acceptance of the by-laws of Coronavirus Committee, and issuing the closures of sacred shrines and the suspension of Friday prayer (7).

In regard to the second dimension, the health system capacity and service coverage of Iran have a suitable condition with 65 schools/universities of medical sciences integrated with health services as the unique country in the world is responsible for covering the whole people's needs. In Iran, where measures have been taken regarding the Primary Health Care (PHC) since 5 years before the Alma-Ata Declaration (1978) the use of the network system is considered as one of the main mechanisms of coping with COVID-19 in a ratified the health system. However, upon the prevalence of Covid-19 in the country, much potential was ignored, one of which was the capacity of the PHC system with ~21,500 centers in rural areas and ~8,000 health centers in the governmental sector. However, after a while, part of the outpatient management protocol was assigned to this extensive network for home-to-home screening and the information on the health status of all Iranians was registered in a system. Therefore, it is possible to follow up on individuals by having access to the patients' contacts and other information (8).

In addition, not assigning epidemiologists at the right time to determine indicators such as fatality and mortality was among the weaknesses that disturbed predictions for estimating care and diagnostic needs. Although many research centers in the country have begun to develop high-sensitivity and specificity diagnostic kits and these experiments are carried out in 50 laboratories, it is still not possible to perform tests on all potential cases. On the other hand, the dissemination of viruses firstly began in the central regions of the country and then intensively spread to other regions (9). The sudden increase in the number of cases led to a shortage of hospital beds in the referral hospitals initially dedicated to these patients, although there are ~130,000 private and public hospital beds in Iran.

This shortage, which has been a concern of the authorities in all provinces, has led to the establishment of care centers after the early discharge of patients from referral hospitals or outpatient admission prior to hospital admission. Although the establishment of these centers took place in the middle of March, with the launch of Command Headquarters, it was attempted to refer patients to these centers after the acute period of the disease was passed if they could not be discharged to home, or to become a center for mild patients. An interesting point in the management of these centers is the combination of military staff and volunteer or hospital personnel that may be somewhat different from international standards as these centers should apparently be managed by military forces to prevent hospital personnel from being separated from their workplace.

On the contrary to the above weaknesses, the diagnosis, treatment, and follow-up of symptomatic and infected patients have been free from the very beginning. A variety of therapeutic and diagnostic protocols have been developed in the form of clinical trials. In this regard, Coronavirus Molecular Diagnostic Network and Anti-Coronavirus Scientific Committee consisting of faculty members of the Iranian universities of medical sciences, and specialists, and experts in various fields with the aim of collaborating with the Ministry of Health and Medical Education (MOHME).

The launch of MOHME online patient screening system for screening more than 75 million people so far and controlling the outbreak was among the effective measures taken to reduce referrals to health centers and reduce the risk of infection in healthy people, of which 146,000 were discovered and referred to health centers (10).

As the third dimension, culture has always been considered one of the effective factors on health (11), the importance of which is particularly clear in the COVID-19 pandemic. The Iranian New Year's celebration (March 21th) is thousands of years old symbolizing renewal in all aspects for Iranians. Therefore, all people prepare for Nowruz from the middle of February which is apparent from the high traffic and crowds of people walking in the streets and all parts of cities. On the other hand, the nearly 15-day holiday of Nowruz is a time for Iranians to make many trips. Thus, the concurrency of COVID-19 pandemic with these days, which happened similarly in China, led to possibly the highest rate of interpersonal contact in the community, and the city-to-city spread of the disease by Nowruz travelers. Although there is a lack of cooperation and attention to the health guidelines by some people, the cooperation of many other members of the community is exemplary. The adherence to the slogan “We stay at home” and avoiding social interactions, performing volunteer activities such as the disinfection of public areas, the voluntary presence at patients' bedside, good-doers' helping provide and produce protective equipment such as scrubs and masks, changing factory production lines to manufacture and prepare disinfectants, gloves, etc., landlords' not receiving rental fees, and obeying the Supreme Leader's orders not to visit sacred shrines and sanctuaries are examples of the culture of sacrifice among Iranian (12).

The last point is that the information provided by Iranian authorities is always regarded as the most reliable information unless, for some reason, this transparency is compromised. Despite the daily reports of the number of the infected, recovered cases and deaths by the MOHME, the negative propaganda in foreign media and cyberspace has been able to effectively worsen the community's attitude toward the Iranian management. This negative wave targets a wide range of issues from the number of deaths reported by the authorities to the news of digging mass graves for the victims and even suggests Iran as the center for spreading the disease through its international airports, even in cities with no airports. Everything considered, while disturbing public opinion, this will lead to distrust toward Iran's effectiveness in dealing with the outbreak in this country (13).

Overall, what has helped Iran control the disease so far can be summarized in several factors: the managerial concept all governance, although delayed, was strongly implemented were religious leaders along with military forces and civil volunteers accompanied the MOHME. On the other hand, the powerful PHC infrastructure and therapeutic, care, and specialized workforce which is appropriately distributed, due to the spread of the universities of medical sciences all across the country, have played important roles in disease management. Despite the actions taken to create an atmosphere of distrust, the honesty of the authorities even in regard to the shortage of resources and equipment is considered an advantage in Iran. And finally, given Iran's specific circumstances, the focus should be put on domestic production, rather than importing equipment, to soon change the country into an exporter of health goods.

In regard with the weaknesses of the system in dealing with the disease, due to the shortage of diagnostic kits at the onset of the disease in the country and its impact on the infected cases and subsequent deaths, some contradictory statistics have been presented which have led to the misinterpretation of the statistics and influenced planning for hospital beds, and hospitalization and patient care facilities. The shortage of data, the epidemiologists' lack of engagement in investigating the disease trend, and presenting different scenarios have also contributed to this matter. The lack of personal protective equipment for the frontline staff and people is also a challenge for the health system that has resulted in the death of a number of physicians and nurses. Furthermore, the lack of advanced equipment for the care of critically ill patients in intensive care units, as a result of sanctions, is what requires to be managed.

In summary regarding the detection of new cases and rapid responses health authorities did the best by supporting people but tracing the cases were not in an appropriate status, they asked them to stay home by family responsibility, but it did not work in some cases and the infected cases were in touch with the public. We did not use the temporary care centers as a part of the PHC facility, social distancing was were supported by other stakeholders not managing by health managers, which may not be promising effective in the future.

Ultimately, it is certain that the COVID-19 pandemic will end as did all previous ones, although it obviously will not be the last. Therefore, the lessons learned from managing it in each country and sharing it with other countries can help prepare the world to deal with future pandemics.

## Data Availability Statement

All datasets presented in this study are included in the article/supplementary material.

## Author Contributions

All authors listed have made a substantial, direct and intellectual contribution to the work, and approved it for publication.

## Conflict of Interest

The authors declare that the research was conducted in the absence of any commercial or financial relationships that could be construed as a potential conflict of interest.

## References

[1] Zhan C Tse C Fu Y Lai Z Zhang H Modeling and prediction of the 2019 coronavirus disease spreading in china incorporating human migration data. medRxiv. (2020). 10.1101/2020.02.18.20024570 PMC7591076 33108386

[2] Coronavirus Cases Available online at: https://www.worldometers.info/coronavirus/country/iran/

[3] Prevention UCfDCa Coronavirus Disease 2019 (Covid-19). (2020). Available online at: https://www.cdc.gov/coronavirus/2019-nCoV/summary.html

[4] Verheul E van de Pas R Health is a political issue. Glob Med. (2010) 27–9. Available online at: https://www.researchgate.net/publication/280729652_Health_is_a_political_issue

[5] Takian A Raoofi A Kazempour S. COVID-19 battle during the toughest sanctions against Iran. Lancet. (2020) 395:1035–6. 10.1016/S0140-6736(20)30668-1 32199073 PMC7138170

[6] Al Shorbaji N. e-health in the Eastern Mediterranean Region: a decade of challenges and achievements. East Mediterr Health J. (2008) 14(Suppl.):S157–73. Available online at: http://applications.emro.who.int/emhj/14_s1/14_s1_s157.pdf 19205616

[7] Thanks to the Supreme Leader for the Efforts of Physicians and Nurses in the Fight Against the Corona Virus (2020). Available online at: http://farsi.khamenei.ir/news-content?id=45033

[8] Yazdi Feyzabadi V Emami M Mehrolhassani MH. Health information system in primary health care: the challenges and barriers from local providers' perspective of an area in Iran. Int J Prev Med. (2015) 6:57. 10.4103/2008-7802.160056 26236444 PMC4505398

[9] Education MoHaM Daily Statistics of Covid-19 in Iran. (2020). Available online at: http://webda.behdasht.gov.ir/index.jsp?siteid=1&fkeyid=&siteid=1&pageid=54782&newsview=201112

[10] Education MoHaM People's Screening System. (2020). Available online at: https://salamat.gov.ir/

[11] Hernandez M Gibb JK. Culture, behavior and health. Evolut Med Public Health. (2019) (2019) 2020:12–3. 10.1093/emph/eoz036 31976074 PMC6970345

[12] University-Industry Cooperation a Mechanism to Deal With the Corona Virus. Available online at: https://www.irna.ir/news/83704872/

[13] Tuite A Bogoch I Sherbo R Watts A Fisman D Khan K. Estimation of COVID-2019 burden and potential for international dissemination of infection from Iran. medRxiv. (2020). 10.1101/2020.02.24.20027375 32176272 PMC7081176

